# Cutaneous manifestations of human T-cell lymphotrophic virus type-1-associated adult T-cell leukemia/lymphoma: retrospective study in Martinique

**DOI:** 10.1186/1742-4690-12-S1-P78

**Published:** 2015-08-28

**Authors:** Emilie Baubion, Mylène Vestris, Jean-Côme Meniane

**Affiliations:** 1 Dermatology, CHU Martinique, Martinique, France; 2 UF 1441 Canceroly Research, CHU Martinique, Martinique, France; 3 Hematology, CHU Martinique, Martinique, France

## Background

Adult T cell leukemia/lymphoma (ATLL) is an aggressive lymphoma that occurs in 2–5% of HTLV-1 infected persons after several decades. Cutaneous manifestations often reveal or accompany the diagnosis of ATLL patients. No data exist regarding cutaneous involvement of adult T-cell leukemia/lymphoma (ATLL) in Martinique. We sought to describe skin involvement characteristics at onset of ATLL in Martinique.

## Methods

We retrospectively studied newly diagnosed ATLL patients admitted to the Hematology and Dermatology Departments of Fort-de-France Hospital during 6-year period (2010-2014) for which skin involvement was proved by the presence of skin lesions with histopathologic confirmation based on morphological and phenotyping.

## Results

The study population included 14 cases (5 men and 9 women). The mean age was 64 years [49-85]. According to the Shimoyama classification, acute subtype was most frequent (n=6, 43 %), followed by lymphoma (n=3, 22 %), chronic (n=2, 14 %) and smoldering subtypes (n=3, 22 %). All patients were HTLV-1 positive serology and had CD3+/CD4+/CD8-/CD7-/CD25+ lymphocyte either on biopsy and, or immunophenoting of blood tumour cell. On upset ATLL skin appears in various forms (see figures). Pleimorphic lesions were commun (n=10, 71%). The most common form were papules (n=10, 71%), nodules (n=5, 36%), squamous lesions (n=4, 29%), erythematous macules (n=3, 21 %) and plaques (n=2, 14%). Less commonly, the lesions may appear as necrotic ulcerations, vesicles or livedo with skin induration. Most of the patients (n=13, 93%) had skin lesions in the trunk and the limbs and 4 of them (31%) had lesions in face, ears or neck. One patient had lesions limited in the face and neck.

## Conclusion

This retrospective study focus on cutaneous manifestations at ATLL diagnosis. Beside classical disseminated papules and nodules, lesions were very polymorphic sometimes micmiking mycosis fungoïde and vascularitis lesions. ATLL diagnosis should be evoked in front of every kind of cutaneous lesion in HTLV-1 endemic area and familiarity with such clinical features may aid in early diagnosis. There was no direct link between the form of lesions and the subtype of ATLL in our study.

